# Ovarian Seminoma

**Published:** 1913-11

**Authors:** 


Ovarian Seminoma. P. Masson, *Bull. et Mem. de la Soc. Anat. de Paris,* November, 1912. Although long mistaken for round cell sarcoma, this tumor is identical with the structure of the testis and cannot be distinguished from testicular seminoma. The tumor is usually massive, yellowish white or rose white, the hardness depending on the amount of connective tissue. The author showed five cases of ovarian and two of testicular seminoma.

